# Une complication rare du kyste hydatique du foie: la pancréatite aiguë

**DOI:** 10.11604/pamj.2015.21.247.7600

**Published:** 2015-08-06

**Authors:** Ammar Mahmoudi, Khadija Zouari

**Affiliations:** 1 Service de Chirurgie Générale et Digestive, CHU Fattouma Bourguiba de Monastir, Monastir, Tunisie

**Keywords:** Pancréatite aiguë, kyste hydatique du foie, fistule kysto-biliaire, échographie, tomodensitométrie, chirurgie, Pancréatite aiguë, kyste hydatique du foie, fistule kysto-biliaire, échographie, tomodensitométrie, chirurgie

## Image en medicine

La pancréatite aiguë (PA) d'origine hydatique est une complication rare du kyste hydatique du foie (KHF). Sa pathogénie relève, comme les PA d'origine biliaire, d'un mécanisme canalaire avec passage de matériel parasitaire à travers une fistule kystobiliaire, suivi d'une obstruction transitoire de la papille avec reflux de bile, mélangée ou non à du liquide intrakystique, dans le canal de Wirsung. Le diagnostic est fondé essentiellement sur l'imagerie médicale. Le traitement des PA d'origine hydatique associe, en plus du traitement symptomatique de la PA et de l'angiocholite éventuellement associée, le traitement de la localisation hydatique hépatique à celui de la fistule biliokystique, et doit assurer la vacuité de la voie biliaire principale (VBP). Nous rapportons l'observation d'un patient de 41 ans, sans antécédents admis pour épigastralgie. La biologie a montré une lipasémie à 8 fois la normale, une cytolyse et une cholestase. L’échographie et la TDM abdominale ont objectivé un KHF des segments VIII et I, affaissé ouvert dans les voies biliaires, avec dilatation de la VBP à 11mm dont le contenu est dense hétérogène compatible avec un matériel hydatique et un aspect de pancréatite aiguë stade B. La vésicule biliaire était alithiasique. Le patient est opéré par sous-costale droite; le kyste avait un périkyste épais, multivésiculaire contenant une fistule biliaire. Il a été réalisé une cholédocotomie, extraction du matériel hydatique, et fermeture sur un drain de Kehr, une fermeture de la fistule kysto-biliaire, une résection du dôme saillant et drainage de la cavité résiduelle. Les suites opératoires étaient simples.

**Figure 1:**
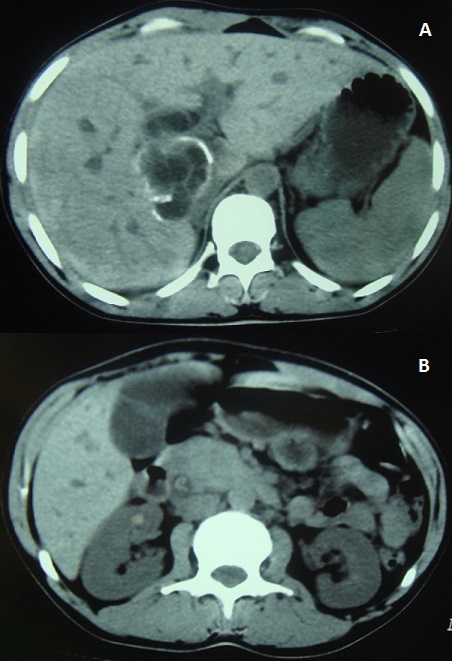
(A) tomodensitométrie abdominale en coupe axiale montrant la présence d'un kyste hydatique du foie des segments VIII et I affaissé, à paroi partiellement calcifiée et ouvert dans les voies biliaires. Les voies biliaires intra-hépatiques sont dilatées; (B) tomodensitométrie abdominale en coupe axiale montrant la dilatation des voies biliaires intra-hépatiques. La voie biliaire principale dans sa portion intra-pancréatique est dilatée renfermant du matériel dense hétérogène compatible avec un matériel hydatique. La tête et l'ischme pancréatiques sont tuméfiés (aspect de pancréatite aiguë stade B)

